# A Formalization of SQL with Nulls

**DOI:** 10.1007/s10817-022-09632-4

**Published:** 2022-07-27

**Authors:** Wilmer Ricciotti, James Cheney

**Affiliations:** Laboratory for Foundations of Computer Science, University of Edinburgh, 10 Crichton St, Edinburgh, EH8 9AB UK

**Keywords:** SQL, Nulls, Semantics, Formalization, Coq

## Abstract

SQL is the world’s most popular declarative language, forming the basis of the multi-billion-dollar database industry. Although SQL has been standardized, the full standard is based on ambiguous natural language rather than formal specification. Commercial SQL implementations interpret the standard in different ways, so that, given the same input data, the same query can yield different results depending on the SQL system it is run on. Even for a particular system, mechanically checked formalization of all widely-used features of SQL remains an open problem. The lack of a well-understood formal semantics makes it very difficult to validate the soundness of database implementations. Although formal semantics for fragments of SQL were designed in the past, they usually did not support set and bag operations, lateral joins, nested subqueries, and, crucially, null values. Null values complicate SQL’s semantics in profound ways analogous to null pointers or side-effects in other programming languages. Since certain SQL queries are equivalent in the absence of null values, but produce different results when applied to tables containing incomplete data, semantics which ignore null values are able to prove query equivalences that are unsound in realistic databases. A formal semantics of SQL supporting all the aforementioned features was only proposed recently. In this paper, we report about our mechanization of SQL semantics covering set/bag operations, lateral joins, nested subqueries, and nulls, written in the Coq proof assistant, and describe the validation of key metatheoretic properties. Additionally, we are able to use the same framework to formalize the semantics of a flat relational calculus (with null values), and show a certified translation of its normal forms into SQL.

## Introduction

SQL is the standard query language used by relational databases, which are the basis of a multi-billion dollar industry. SQL’s semantics is notoriously subtle: the standard (ISO/IEC 9075:2016) uses natural language that implementations interpret in different ways.

Relational databases are the world’s most successful example of declarative programming. Commercial databases optimize queries by applying rewriting rules to convert a request into an equivalent one that can be executed more efficiently, using the database’s knowledge of data organization, statistics, and indexes. However, the lack of a well-understood formal semantics for SQL makes it very difficult to validate the soundness of candidate rewriting rules, and even widely used database systems have been known to return incorrect results due to bugs in query transformations (such as the “COUNT bug”) [10, 14]. As a result, many database systems conservatively use a limited set of very well-understood rewrite rules.

An accurate understanding of the semantics of SQL is also required to validate techniques used to integrate SQL queries in a host programming language. One such technique, which has been particularly influential in recent years, is *language-integrated query*: it is based on a domain specific sublanguage of the host programming language, whose expressions can be made to correspond, after some manipulation, to SQL queries. In order for the validity of this correspondence to be verified, we need a formal semantics of SQL.

One of SQL’s key features is incomplete information, i.e. *null values*. Null values are special tokens that indicate a “missing” or “unknown” value. Unlike the “none” values in “option” or “maybe” types in functional languages such as ML, Haskell, or Scala, null values are permitted as values of any field by default unless explicitly ruled out as part of a table’s schema (type declaration). Moreover, standard arithmetic and other primitive operations are extended to support null values, and predicates are extended to three-valued interpretations, to allow for the possibility that a relationship cannot be determined to be either true or false due to null values. As a result, the impact of nulls on the semantics of SQL is similar to that of *effects* such as null pointers, exceptions, or side-effecting references in other programming languages: almost any query can have surprising behavior in the presence of nulls.

SQL’s idiosyncratic treatment of nulls is a common source of bugs in database applications and query optimizers, especially in combination with SQL’s *multiset* (or *bag*) semantics. For example, consider the following three queries: 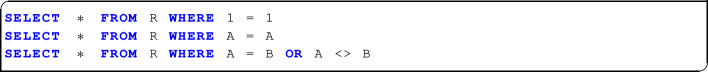 over a relation *R* with fields *A*, *B*. In conventional two-valued logic, all three queries are equivalent because the -clauses are tautologies. However, in the presence of nulls, all three queries have different behavior: the first simply returns *R*, while the second returns all elements of *R* whose *A*-field is nonnull, and the third returns all elements of *R* such that both *A* and *B* values are nonnull. In the second query, if a record’s *A* value is null, then the truth value of $A = A$ is *maybe*, and such records are not included in the resulting set. Likewise, if one of *A* or *B* (or both!) is null, then $A = B \vee A \ne B$ has truth value *maybe*.

This problem, unfortunately, pervades most SQL features, even ones that do not explicitly refer to equality tests. For example, in the absence of null values, Guagliardo and Libkin observe that all three of the following queries have equivalent behavior ( [13]): 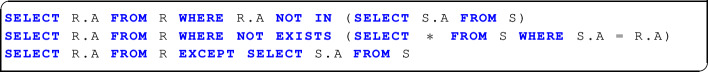 but all three have *different* behavior when presented with the input table $R = \{1,null\}$ and $S = \{null\}$. The first results in $\emptyset$, the second in $\{1,null\}$, and the third in $\{1\}$.

SQL’s rather counterintuitive semantics involving NULLs and three-valued logic leads query optimizers to be conservative in order to avoid subtle bugs. Database implementations tend to restrict attention to a small set of rules that have been both carefully proved correct (on paper) and whose correctness has been validated over time. This means that to get the best performance, a programmer often needs to know what kinds of optimizations the query optimizer will perform and how to reformulate queries to ensure that helpful optimizations take place. Of course, this merely passes the buck: now the programmer must reason about the correctness or equivalence of the more-efficient query, and as we have seen this is easy to get wrong in the presence of nulls. As a result, database applications are either less efficient or less reliable than they should be.

Formal verification and certification of query transformations offers a potential solution to this problem. We envision a (not too distant) future in which query optimizers are *certified*: that is, in addition to mapping a given query to a hopefully more efficient one, the optimizer provides a checkable proof that the two queries are equivalent. Note that (as with certifying compilers [15]) this does not require proving the correctness or even termination of the optimizer itself. Furthermore, we might consider several optimizers, each specializing in different kinds of queries.

Before we get too excited about this vision, we should recognize that there are many obstacles to realizing it. For example, before we can talk about proving the correctness of query transformations, let alone mechanically checked proofs, we need to have a suitable semantics of queries. Formal semantics for SQL has been investigated intermittently, including mechanized formalizations and proofs; however, most such efforts have focused on simplified core languages with no support for nulls [3, 6, 18], meaning that they can and do prove equivalences that are false in real databases, which invariably do support nulls (a recent exception to this is $\textit{SQL}_{\textit{Coq}}$ [2], which we will discuss later). Part of the reason for neglecting nulls and three-valued logic is that the theory of relational databases and queries has been developed largely in terms of the *relational algebra* which does not support such concepts. Recent work by Guagliardo and Libkin [13] provides the first (on-paper) formal semantics of SQL with nulls (we will call this $\textsf {Null}{} \textit{SQL}$). $\textsf {Null}{} \textit{SQL}$ is the first formal treatment of SQL’s nulls and three-valued semantics, and it has been validated empirically using random testing to compare with the behaviour of real database engines, but mechanized formalizations of the semantics of SQL with nulls have only appeared recently.

### Contributions

This paper is a report about our formalization of SQL with null values, three-valued logic, and lateral joins: our development can be publicly accessed at its GitHub repository (https://github.com/wricciot/nullSQL). The most complete formalization of SQL to date is $\textit{SQL}_{\textit{Coq}}$ [2], which was developed concurrently with our work: it formalizes a variant of $\textsf {Null}{} \textit{SQL}$ with grouping and aggregates and a corresponding bag-valued relational algebra, proving the equivalence between the two. Our work does not deal with grouping and aggregation; however, it does provide a more accurate formalization of well-formedness constraints for SQL expressions. The well-formedness judgment defined in $\textit{SQL}_{\textit{Coq}}$ accepts queries using free attribute names (not bound to an input table), which are rejected by concrete implementations; in the formalization, such queries are assigned a dummy semantics in the form of default values.

Another relevant formalization is $\textsf {HoTT}{} \textit{SQL}$ by Chu et al. [6], which does not allow incomplete information in tables; as it turns out, formalizing SQL with nulls requires us to deal with issues that are not immediately evident in $\textsf {HoTT}{} \textit{SQL}$, and thus provides us with an opportunity to consider alternatives to some of their design choices.

We summarize here the key features of our formalization compared to the existing work.

*Representation of tables.* The $\textsf {HoTT}{} \textit{SQL}$ paper describes two concrete alternatives for the representation of tables: the list model and the *K*-relation model [12]. They argue that lists are difficult to reason on because of the requirement that they be equal up to permutation of elements, and that*K*-relations require the invariant of finite-supportedness to be wired through each proof. They then go on to extend the *K*-relation model to *K* allowing infinite cardinalities (through HoTT types) and claim this is a substantial improvement; they also use univalent types ${\mathbf {0}}$ and ${\mathbf {1}}$ to represent truth values. However, they do not prove an adequacy property relating this representation to a conventional one. Despite the ease of reasoning with the $\textsf {HoTT}{} \textit{SQL}$ approach, it is unclear how to adapt it to three-valued logic.

As for $\textit{SQL}_{\textit{Coq}}$, [2] does not discuss the representation of tables in great detail; however, their formalization uses a bag datatype provided in a Coq library.

In this paper, we show instead that the difficulty of reasoning on lists up to permutations, which partly motivated the recourse to HoTT, is a typical proof-engineering issue, stemming from a lack of separation between the properties that the model is expected to satisfy, and its implementation as data (which is typical of type theory). Our key contribution is, therefore, the definition of *K*-relations as an abstract data type whose inhabitants can only be created, examined, and composed by means of structure-preserving operations, and its concrete implementation as normalized lists.

*Reasoning on relations.* This is a related point. Reasoning on an ADT cannot use the case analysis and induction principles that are normally the bread and butter of Coq users; for this reason, our ADT will expose some abstract well-behavedness properties that can be used as an alternative to concrete reasoning. Additionally, we will assume heterogeneous (*“John Major”*) equality to help with the use of dependent types, and functional extensionality to reason up to rewriting under binders (such as the $\varSigma$ operator of *K*-relations expressing projections – and more complex maps in our formalization).

*The formalized fragment of SQL.* Aside from nulls, there are several differences between the fragments of SQL used by the three formalizations. To list a few:

- $\textsf {HoTT}{} \textit{SQL}$ does not employ names at any level, therefore attributes must be referenced in a de Bruijn-like style, by position in a tuple rather than by name; $\textit{SQL}_{\textit{Coq}}$ uses names for attributes, but not for tables, and relies on the implicit assumption that attributes be renamed so that no aliasing can happen in a cross product; in our formalization, names are used to reference attributes, and de Bruijn indices to reference tables; our semantics is nameless.
- Since $\textsf {HoTT}{} \textit{SQL}$ does not have names, it does not allow attributes to be projected just by referencing them in a select clause (as we do), but it provides additional language expressions to express projections as a (forgetful) reshuffling of an input sequence of attributes.
- $\textit{SQL}_{\textit{Coq}}$, on the other hand, by assuming that no attribute clash can occur, does not address the attribute shadowing problem mentioned by [13].
- Both $\textsf {HoTT}{} \textit{SQL}$ and $\textit{SQL}_{\textit{Coq}}$ do consider *grouping* and *aggregation* features, which are not covered by [13], nor by our formalization;
- Unlike both $\textsf {HoTT}{} \textit{SQL}$ and $\textit{SQL}_{\textit{Coq}}$, we formalize SQL queries with  input, introduced in the SQL:1999 standard and supported by recent versions of DBMSs such as Oracle, PostgreSQL, and MySQL. When a subquery appearing in the  clause is preceded by , that subquery is allowed to reference attributes introduced by the preceding  items: this means that while normally the  items of a  query are evaluated independently, a  subquery needs to be evaluated once for every tuple in the preceding  items, making its semantics substantially more complicated.

*Boolean semantics vs. three-valued semantics.* As we mentioned above, in $\textsf {HoTT}{} \textit{SQL}$ the evaluation of the  clauses of queries yields necessarily a Boolean value. However, in standard SQL, conditional expressions can evaluate to an uncertain truth value, due to the presence of incomplete information in the data base. The lack of an obvious denotation of the uncertain truth value as a HoTT type makes it challenging to extend that work to nulls even in principle. Our formalization, like Benzaken and Contejean’s, provides a semantics for $\textsf {Null}{} \textit{SQL}$ based on three-valued logic; additionally, we provide a Boolean semantics as well: we can thus formally derive Guagliardo and Libkin’s proof that, even in the presence of nulls, three-valued logic does not increase the expressive power of SQL, and even extend it to queries with  input. Whether such a property holds in the presence of grouping and aggregation does not appear to have been investigated.

*Relational calculus vs. SQL.* The language-integrated query feature of programming languages such as Kleisli [26], Links [8], and Microsoft’s C# and F# allows a user to express database queries in a typed domain-specific sublanguage which blends in nicely with the rest of the program. Core calculi such as the nested relational calculus [5] () have been used to provided a theoretical basis to study language-integrated query: in particular, Wong’s conservativity theorem ( [25]) implies that every  query mapping flat tables to flat tables can be *normalized* to a flat relational calculus query, not using nested collections as intermediate data. Such flat queries correspond closely to SQL queries, and it is straightforward to give an algorithm to translate the former into the latter. Furthermore, in [20] and [22], we extended  to allow queries mixing set and bag collections, and we noted that in this language, under additional conditions, it is still possible to normalize flat queries to a form that directly corresponds to SQL, as long as  inputs are allowed.

However, the correspondence established by these works is rather informal: the correctness of translations from  to SQL has not been proved formally, at least to our knowledge. In Sect. 8, we fill this gap in the literature: we formally define flat relational calculus normal forms using sets and bags and their semantics, show a translation mapping them to SQL, and prove that the translation preserves the semantics of the original query.

### Structure of the Paper

We start in Sect. 3 by describing our formalization of the syntax of $\textsf {Null}{} \textit{SQL}$, discussing our implementation choices and differences with the official SQL syntax; Sect. 4 is devoted to our semantic model of relations, particularly its implementation as an abstract data type; in Sect. 5, we describe how SQL queries are evaluated to semantic relations, using both Boolean and three-valued logic; Sect. 7 formalizes Guagliardo and Libkin’s proof that the two versions of the semantics have the same expressive power; finally Sect. 8 gives a semantics of normalized flat relational calculus terms and gives an algorithm to translate them to SQL queries, proving its correctness.

## Overview of the Formalization

The formalization we describe is partitioned in several modules and functors. In some cases, these serve as little more than namespaces, or are used mostly for the purpose of presentational separation. For example, the various parts of this development are defined in terms of an underlying collection of named tables, namely *the data base* *D*; rather than cluttering all the definitions with references to *D* and its properties, we package their signature in a module type DB and assume that a concrete implementation is given.

The syntax of $\textsf {Null}{} \textit{SQL}$, including rules defining well-formedness of queries and other expressions, is defined in a module of type SQL.

## Syntax

We formalize a fragment of SQL consisting of select-from-where queries (including “select-star”) with correlated subqueries connected with  and  and operations of union, intersection and difference. Both set and bag (i.e. multiset) semantics are supported, through the use of the keywords  and . We assume a simple data model consisting of constants ${\mathbf {k}}$ along with the unspecified  value. We make no assumption over the semantics of constants, which may thus stand for numeric values, strings, or any other kind of data; however, for the purpose of formalization it is useful to assume that the constants be linearly ordered, for example by means of the lexicographic order on their binary representation. Relations are bags of *n*-tuples of values, where *n* is the arity of the relation. Our syntax is close to the standard syntax of SQL, but we make a few simplifying changes:

- The tables in the  clause of  queries are referenced by a 0-based de Bruijn index rather than by name; however, attributes are still referenced by name.
- Attribute (re)naming using , both in  and , is mandatory.
- The  clause is mandatory ( must be used when no condition is given).
- An explicit syntax ($\textit{table}~x$ or $\textit{query}~Q$) is provided to differentiate between tables stored by name in the database and tables resulting from a query.

Hence, if *R* is a relation with column names *A*, *B*, *C*, the SQL query  must be expressed as  0.$\textit{table}$.

For compactness, we will write  as a colon “:”. The full syntax follows:The  clause of a query takes a list of terms, which include null or constant values, and references to attributes one of the tables in the form *n*.*x*, where *n* is the index referring to an input relation in the  clause, and *x* is an attribute name. The input of the query is expressed by the  clause, which references a *generator* *G* consisting of a sequence of *frames* separated by the  keyword; each frame is a sequence $\overrightarrow{T:\sigma }$ of input tables paired with a schema (allowing attribute renaming); an input table can be defined using variables introduced in a previous frame, but not in the same frame; concretely, in a query: 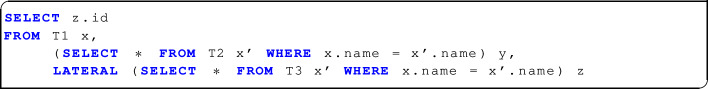 the expression introducing the variable  is ill-formed, because it uses the variable , which is introduced in the same frame; however, the very similar expression associated to  is well-formed, because it is part of a different frame introduced by . In our Coq formalization, we will model frames as lists, and sequences of frames as lists of lists.

Conditions for the  clause of queries include Booleans and Boolean operators (, , , , ), comparison of conditions with , comparison of terms with , membership tests for tuples (), non-emptiness of the result of subqueries (), and custom predicates $P^n(\overrightarrow{t_n})$ (where $P^n$ is an *n*-ary Boolean predicate, and $\overrightarrow{t_n}$ an *n*-tuple of terms).

The abstract syntax we have presented in Sect. 3 is made concrete in Coq by means of inductive types. 
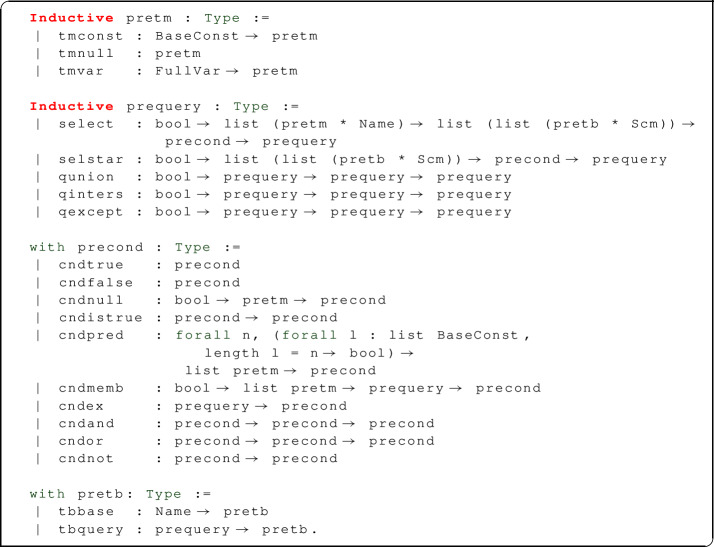
 Query constructors select and selstar take a Boolean argument which, when it is true, plays the role of a  selection query; similarly, the Boolean argument to constructors qunion, qinters, and qexcept plays the role of the  modifier allowing for union, intersection, and difference according to bag semantics. Conditions using base predicates are expressed by the constructor cndpred: notice that we do not formally specify the set of base predicates defined by SQL, but allow any *n*-ary function from constant values (of type BaseConst) to Booleans expressible in Coq to be embedded in an SQL query: such functions can easily represent SQL predicates including equality, inequality, numerical “greater than” relations,  on strings, and many more.

**Fig. 1:**
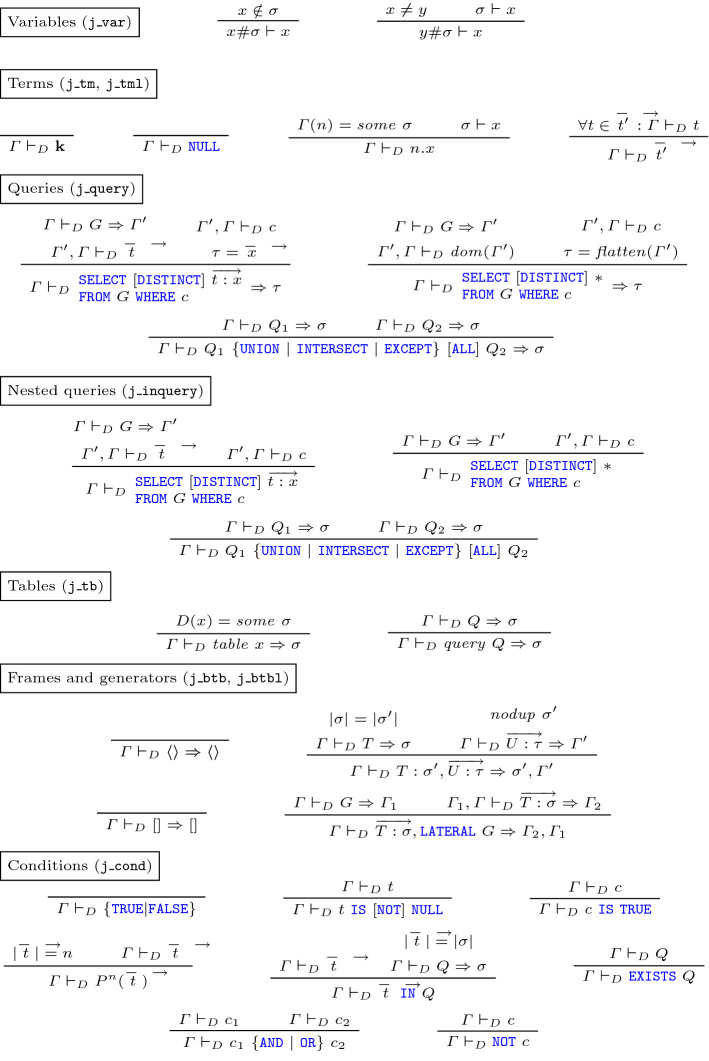
Well-formed SQL syntax

We use well-formedness judgments (Fig. 1) to filter out meaningless expressions, in particular those containing table references that cannot be resolved because they point to a table that is not in the  clause, or because a certain attribute name is not in the table, or is ambiguous (as it happens when a table has two columns with the same name). The formalization of legal SQL expressions has mostly been disregarded in other work, either because the formalized syntax was not sufficiently close to realistic SQL ($\textsf {HoTT}{} \textit{SQL}$ does not use attribute or table names), or because it was decided to assign a dummy semantics to illegal expressions (as in $\textit{SQL}_{\textit{Coq}}$).

There are distinct judgments for the well-formedness of attribute names and terms, and five distinct, mutually defined judgments for tables, frames, generators, conditions, queries and existentially nested queries. Each judgment mentions a context $\varGamma$ which assigns a schema (list of attribute names) to each table declared in a  clause. A parameter *D* (*data base*) provides a partial map from table names *x* to their (optional) schema *D*(*x*).

We review some of the well-formedness rules. The rules for terms state that constant literals ${\mathbf {k}}$ and null values are well formed in all contexts. To check whether an attribute reference *n*.*x* is well formed (where *n* is a de Bruijn index referring to a table and *x* an attribute name), we first perform a lookup of the *n*-th schema in $\varGamma$: if this returns some schema $\sigma$, and the attribute *x* is declared in $\sigma$ (with no repetitions), then *n*.*x* is well formed. The rules for conditions recursively check that nested subqueries be well-formed and that base predicates $P^n$ be applied to exactly *n* arguments.

The well-formedness judgments for queries and tables assign a schema to their main argument. Similarly, well-formed frames of tables are assigned the corresponding sequence of schemas, i.e. a context. The well-formedness judgment for generators uses, recursively, the well-formedness of frames, where each frame added to the generator must be well-formed in a suitably extended context (notice that the last frame is added to the left, contrary to SQL syntax, but coherently with Coq’s notation for lists), and finally returns a context obtained by concatenating all the contexts assigned to the individual well-formed frames.

The SQL standard allows well-formed queries to return tables whose schema contains repeated attribute names (e.g. ), but requires attribute references in terms to be unambiguous (so that, if the previous query appears as part of a larger one, the attribute name  can be used, but  cannot). This behaviour is faithfully mimicked in our well-formedness judgments: while well-formed terms are required to only use unambiguous attribute references, the rules for queries do not check that the schema assignment be unambiguous. Furthermore, in a  query that is not contained in an  clause, the star is essentially expanded to the attribute names of the input tables (so that, for example,  is rejected even though the inner query is accepted, and the ambiguous attribute name  is not explicitly referenced).

As an exception, when a  query appears inside an  clause (meaning it is only run for the purpose of checking whether its output is empty or not), SQL considers it well-formed even when the star stands for an ambiguous attribute list. Thus we model this situation as a different well-formedness predicate, with a more relaxed rule for ; furthermore, since the output of an existential subquery is thrown away after checking for non-emptiness, this predicate does not return a schema.

In our formalization, we need to prove weakening only for the term judgment, but not for queries, tables or conditions; weakening for terms is almost painless and only requires us to define a lift function that increments table indices by a given *k*.

Thus, if a term *t* is well-formed in a context $\varGamma$, then it is also well-formed in an extended context $\varGamma ',\varGamma$, provided that we lift it by an amount corresponding to the length of $\varGamma '$.

### Lemma 1

If , then for all $\varGamma '$ we have .

## *K*-Relations as an Abstract Data Type

We recall the notion of *K*-*relation*, introduced in [12] by Green et al.: for a commutative semi-ring $(K,+,\times ,0,1)$ (i.e. $(K,+,0)$ and $(K,\times ,1)$ are commutative monoids, $\times$ distributes over $+$, and 0 annihilates $\times$), a *K*-relation is a *finitely supported* function *R* of type $T \rightarrow K$, where by finitely supported we mean that $R~t \ne 0$ only for finitely many *t* : *T*. *K*-relations constitute a natural model for databases: for example, if $K = {\mathbb {N}}$, *R* *t* can be interpreted as the multiplicity of a tuple *t* in *R*, and finite-supportedness corresponds to the finiteness of bags. In Coq, we can represent *K*-relations as (computable) functions: however, each function must be proved finitely supported separately, cluttering the formalization. To minimize the complication, we model *K*-relations by means of an abstract data type (as opposed to the concrete type of functions); this technique was previously used by one of the authors to formalize binding structures [19].

Just as in the theory of programming languages, an abstract data type for *K*-relations does not provide access to implementation details, but offers a selection of operations (union, difference, cartesian product) that are known to preserve the structural properties of *K*-relations, and in particular finite-supportedness. For the purpose of this work, the ADT we describe is specialized to ${\mathbb {N}}$-relations; we fully believe our technique can be adapted to general commutative semi-rings (including the provenance semi-rings that provided the original motivation for *K*-relations), with some adaptations due to the fact that our model needs to support operations, like difference, that are not available in a semi-ring.

Our abstract type of relations is defined by means of the following signature: 
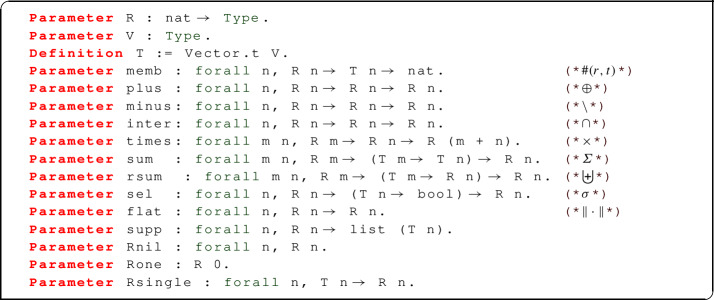
 This signature declares a type family $\mathtt {R~n}$ of $\mathtt {n}$-ary relations, and a type $\mathtt {V}$ of data values. The type family $\mathtt {T~n}$ of $\mathtt {n}$-tuples is defined as a vector with base type $\mathtt {V}$. The key difference compared to the concrete approach is that, given a relation *r* and a tuple *t*, both with the same arity, we obtain the multiplicity of *t* in *r* as $\#(r,t)$, where $\#(\cdot ,\cdot )$ is an abstract operator; the concrete style *r* *t* is not allowed because the type of *R* is abstract, i.e. we do not know whether it is implemented as a function or as something else.

We also declare binary operators $\oplus$, $\backslash$, and $\cap$ for the disjoint union, difference, and intersection on *n*-ary bags. The cartesian product $\times$ takes two relations of possibly different arity, say *m* and *n*, and returns a relation of arity $m+n$.

The operator $\mathtt {sum~r~f}$, for which we use the notation $\sum _r f$ (or, sometimes, $\sum _{x \leftarrow r} f~x$) represents bag comprehension: it takes a relation *r* of arity *m* and a function *f* from *m*-tuples to *n*-tuples, and builds a new relation of arity *n* as a disjoint union of all the *f* *x*, where *x* is a tuple in *r*, taken with its multiplicity; note that for such an operation to be well-defined, we need *r* to be finitely supported. We also provide a more general form of comprehension $\mathtt {rsum~r~g}$, with the notation $\biguplus _r g$ (or, equivalently, $\biguplus _{x \leftarrow r} g~x$), where the function *g* maps *m*-tuples to *n*-relations: the output of this comprehension will be a new relation of arity *n* built by taking the disjoint union of all the relations *g* *x*, where *x* is a tuple in *r*, taken with its multiplicity. Again, this operation is well-defined only if *r* is finitely supported.

Filtering is provided by $\mathtt {sel~r~p}$ (notation: $\sigma _p(r)$), where *p* is a boolean predicate on tuples: this will return a relation that contains all the tuples of *r* that satisfy *p*, but not the other ones.

We also want to be able to convert a bag *r* to a set (i.e. 0/1-valued bag) $\Vert r \Vert$ containing exactly one copy of each tuple present in *r* (regardless of the original multiplicity). Finally, there is an operator $\mathtt {supp~r}$ returning a list of tuples representing the finite support of *r*.

Rnil n identifies the standard empty relation of arity n, and similarly Rone is the standard 0-ary singleton containing exactly one copy of the empty tuple. We also provide Rsingle n t, or the singleton relation containing the tuple t of arity n, although this can easily be defined in terms of Rone and sum.

In our approach, all the operations on abstract relations mentioned so far are declared but not concretely defined. When ADTs are used for programming, nothing more than the signature of all operations is needed, and indeed this suffices in our case as well if all we are interested in is defining the semantics of SQL in terms of abstract relations. However, proving theorems about this semantics would be impossible if we had no clue about what these operations do: how do we know that $\oplus$ really performs a multiset union, and $\cap$ an intersection? To make reasoning on abstract relations possible without access to their implementation, we will require that any implementation shall provide some correctness criteria, or proofs that all operations behave as expected.

The full definition of the correctness criteria for abstract relations as we formalized them in Coq is as follows: 
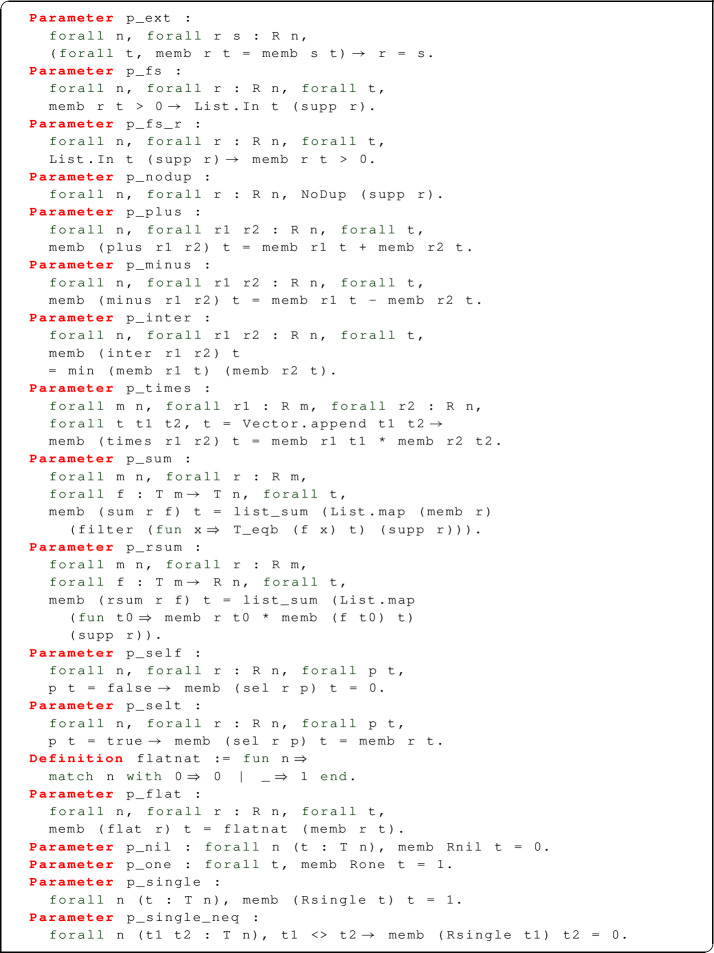
 A first, important property is that relations must be extensional: in other words, any two relations containing the same tuples with the same multiplicities, are equal; this is not true of lists, because two lists containing the same elements in a different order are not equal. Relations should also be finitely supported, and we expect the support not to contain duplicates. The properties for the standard 0-ary relations Rnil and Rone describe the standard 0-ary relations, which implicitly employs the fact that the only 0-tuple is the empty tuple. The properties for plus, minus, inter express the behaviour of disjoint union, difference, and intersection: for instance, a tuple $\#(r \oplus s, t)$ is equal to $\#(r,t) + \#(s,t)$. The behaviour of cartesian products is described as follows: if $r_1$ and $r_2$ are, respectively, an *m*-ary and an *n*-ary relation, and *t* is an $(m+n)$-tuple, we can split *t* into an *m*-tuple $t_1$ and an *n*-tuple $t_2$, and $\#(r_1 \times r_2,t) = \#(r_1,t_1) * \#(r_2,t_2)$. The behaviour of filtering (p_self, p_selt) depends on whether the filter predicate *p* is satisfied or not: $\#(\sigma _p(r),t)$ is equal to $\#(r,t )$ if $p~t = \mathtt {true}$, but it is zero otherwise.

The value of $\#(\Vert r \Vert ,t)$ is one if $\#(r,t)$ is greater than zero, or zero otherwise. Finally, p_sum and p_rsum describe the behaviour of bag comprehensions by relating it to the support of the base relation: $\#(\sum _r~f,t)$ is equal to the sum of multiplicities of those elements *x* of *r* such that $t = f~x$; this value can be obtained by applying standard list functions to $\mathtt {supp}~r$; $\#(\biguplus _r~g,t)$ is equal to the sum of multiplicities of the elements *x* of *r* multiplied by the multiplicities of *t* in *g* *x*.

### A Model of *K*-Relations

The properties of R that we have assumed describe a “naïve” presentation of *K*-relations: they really are nothing more than a list of desiderata, providing no argument (other than common sense) to support their own satisfiability. However, we show that an implementation of R (that is, in logical terms, a model of its axioms) can be given within the logic of Coq.

Crucially, our implementation relies on the assumption that the type V of values be totally ordered under a relation $\le _\mathtt {V}$; consequently, tuples of type T n are also totally ordered under the corresponding lexicographic order $\le _\mathtt {T~n}$. We then provide an implementation of R n by means of a refinement type: 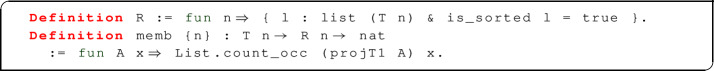 where is_sorted l is a computable predicate returning true if and only if l is sorted according to the order $\le _\mathtt {T~n}$. The inhabitants of R n are dependent pairs $\langle l,H \rangle$, such that $l : \mathtt {T~n}$ and $H : \mathtt {is\_sorted}~l = \mathtt {true}$. The multiplicity function for relations memb is implemented by counting the number of occurrences of a tuple in the sorted list (count_occ is a Coq standard library function on lists).

The most important property that this definition must satisfy is extensionality. For any two sorted lists $l_1, l_2$ of the same type, we can indeed prove that whenever they contain the same number of occurrences of all elements, they must be equal: however, to show that $\langle l_1, H_1 \rangle = \langle l_2, H_2 \rangle$ (where $H_i : \mathtt {is\_sorted}~l_i = \mathtt {true}$) we also need to know that the two proofs $H_1$ and $H_2$ are equal. Knowing that $l_1 = l_2$, this is a consequence of uniqueness of identity proofs (UIP) on bool, which is provable in Coq (unlike generalized UIP).

Operations on relations can often be implemented using the following scheme: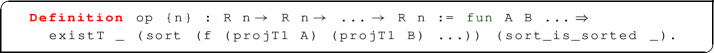 where f is some function of type list (T n) $\rightarrow$ list (T n) $\rightarrow \ldots \rightarrow$ list (T n). Given relations A, B ... we apply f to the underlying lists projT1 A, projT1 B,...; then, we sort the result and we lift it to a relation by means of the dependent pair constructor existT. The theorem sort_is_sorted states that is_sorted (sort l) = true for all lists l. The scheme is used to define disjoint union, difference and intersection:
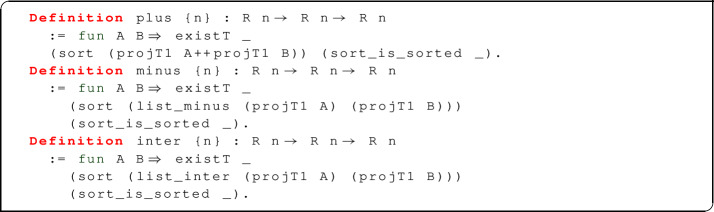
 For disjoint union, f is just list concatenation. For difference, we have to provide a function list_minus, which could be defined directly by recursion in the obvious way; instead, we decided to use the following definition: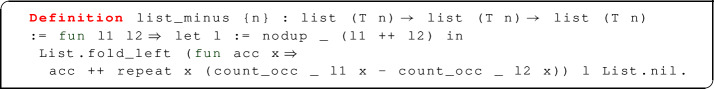 This definition first builds a duplicate-free list l containing all tuples that may be required to appear in the output. Then, for each tuple x in l, we add to the output as many copies of x as required (this is the difference between the number of occurrences of x in l1 and l2). The advantage of this definition is that it is explicitly based on the correctness property of relational difference: thus, the proof of correctness is somewhat more direct. The same approach can be used for intersection and, with adaptations, for cartesian product.

Finally, sum, rsum, sel, and flat reflect, respectively, list map, concat-map, filter, and duplicate elimination.

We do not provide an operation to test for the emptiness of a relation, or to compute the number of tuples in a relation; however, this may be readily expressed by means of sum: all we need to do is map all tuples to the same distinguished tuple. The simplest option is to use the empty tuple $\langle \rangle$ and check for membership:

$$\begin{aligned} \mathtt {card}~S := \#(\sum _S (\lambda x.\langle \rangle ), \langle \rangle ) \end{aligned}$$

The correctness criterion for card, stating that the cardinality of a relation is equal to the sum of the number of occurrences of all tuples in its support, is an immediate consequence of its definition and of the property p_sum:

#### Lemma 2

$$\mathtt {card}~S = \mathtt {list\_sum}~[ \#(S,x) \vert x \leftarrow \mathtt {supp}~S ]$$

## Formalized Semantics

The formal semantics of SQL can be given as a recursively defined function or as an inductive judgment. Although in our development we considered both options and performed some of the proofs in both styles, we will here only discuss the latter, which has proven considerably easier to reason on. As we intend to prove that three-valued logic (3VL) does not add expressive power to SQL compared to Boolean (two-valued) logic (2VL), we actually need two different definitions: a semantic evaluation based on 3VL (corresponding to the SQL standard), and a similar evaluation based on Boolean logic. We factorized the two definitions, which can be obtained by instantiating a Coq functor to the chosen notion of truth value.

### Truth Values

**Fig. 2:**
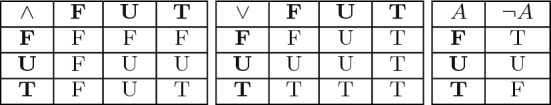
Three-valued logic truth tables

For the semantics of SQL conditions, we use an abstract type ${\mathbf {B}}$ of truth values: this can be instantiated to Boolean values (bool) or to 3VL values (tribool, with values ttrue or $\text {T}$, tfalse or $\text {F}$, and unknown or $\text {U}$): in the latter case, we obtain the usual three-valued logic of SQL. Technically, 3VL refers either to Kleene’s “strong logic of indeterminacy”, or to Łukasiewicz’s L3 logic, which share the same values and truth tables for conjunction, disjunction, and negation (Figure 2); both logics also define an implication connective, with different truth tables: since implication plays no role in the semantics of SQL, it is omitted in our formalization.

For convenience, bool and tribool will be packaged in modules Sem2 and Sem3 of type SEM together with some of their properties. 
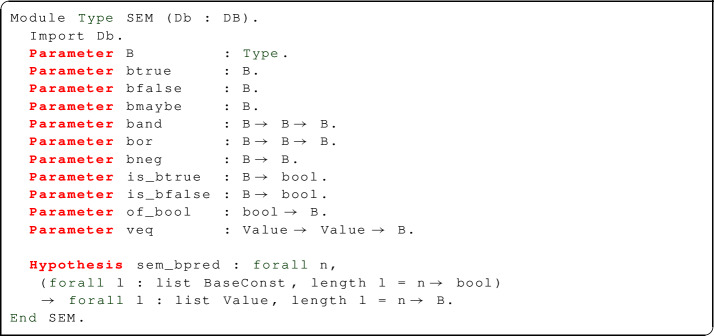
SEM declares the abstract truth values btrue, bfalse, bmaybe (in Sem3, bmaybe is mapped to the uncertain value unknown; in Sem2, both bmaybe and bfalse are mapped to false). SEM also declares abstract operations (band, bor, bneg), operations relating abstract truth values and Booleans (is_btrue, is_bfalse, of_bool), a B-valued equality predicate for SQL values (including NULLs), and an operation sem_bpred which lifts *n*-ary Boolean-valued predicates on constants to B-valued predicates on SQL values (including NULLs): this is used to define the semantics of SQL conditions using base predicates. A theorem sem_bpred_elim describes the behaviour of sem_bpred: if the list of values l provided as input does not contain NULLs, it is converted to a list of constants cl, then the base predicate p is applied to cl; this yields a Boolean value that is converted to B by means of of_bool. If l contains one or more NULLs, sem_bpred will return bmaybe.

### A Functor of SQL Semantics

**Fig. 3:**
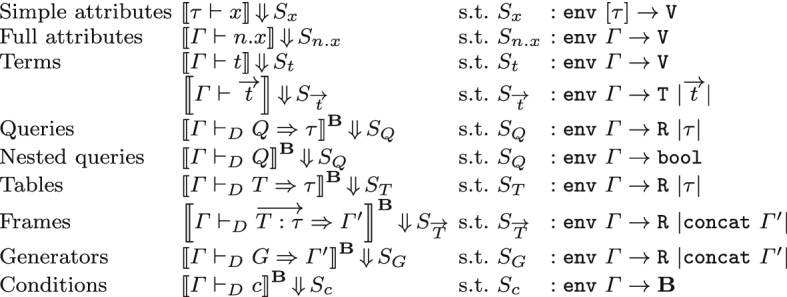
Formal semantics of SQL (types)

In Coq, when defining a collection of partial maps for expressions subject to well-formedness conditions, we can use an “algorithmic approach” based on dependently typed functions, or a “declarative approach” based on inductively defined judgments. The two alternatives come both with benefits and drawbacks; for the purposes of this formalization, consisting of dozens of cases with non-trivial definitions, we judged the declarative approach as more suitable, as it helps decouple proof obligations from definitions. Our inductive judgments implement SQL semantics according to the following style. When a certain expression (query, table or condition) is well-formed for a context $\varGamma$, we expect its semantics to depend on the value assignments for the variables declared in $\varGamma$: we call such an assignment an *environment* for $\varGamma$ (which has type $\mathtt {env}~\varGamma$ in our formalization); thus, we define a semantics that assigns to each well-formed expression an *evaluation*, i.e. a function taking as input an environment, and returning as output a value, tuple, relation, or truth value. Subsequent proofs do not rely on the concrete structure of environments, but internally they are represented as lists of lists of values, which have to match the structure of $\varGamma$: 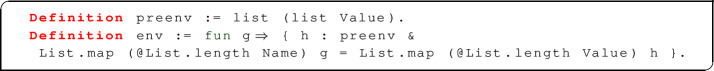 Similarly to well-formedness judgments, we have judgments for the semantics of attribute names and terms, and five mutually defined judgments for the various expression types of SQL. Figure 3 summarizes the judgments, highlighting the type of the evaluation they return. In our notation, we use judgments  with a superscript ${\mathbf {B}}$ denoting their definition can be instantiated to different notions of truth value, in particular, bool and tribool; we will use the notation  and  for the two instances. The semantics of attributes and terms does not depend on the notion of truth value, thus the corresponding judgments do not have a superscript. Concretely, our Coq formalization provides a module Evl for the judgments that do not depend on ${\mathbf {B}}$, and a functor SQLSemantics for the other judgments, which we instantiate with the Sem2 and Sem3 we described in the previous section.

We can prove that our semantics assigns only one evaluation to each SQL expression.

#### Lemma 3

For all judgments ${\mathfrak {J}}$, if  and , then $S=S'$.

Thanks to the previous result, whenever , we are allowed to use the notation  for the semantic evaluation *S*, with no ambiguity.

Simple attributes are defined in a schema rather than a context: their semantics  maps an environment for the singleton context $[\tau ]$ to a value. Similarly, the semantics of fully qualified attributes  maps an environment for $\varGamma$ to a value. In both cases, the output value is obtained by lookup into the environment.

The evaluation of terms  returns a value for *t* given a certain environment $\gamma$ for $\varGamma$. In our definition, terms can be either full attributes *n*.*x*, constants ${\mathbf {k}}$, or NULL. We have just explained the semantics of full attributes; on the other hand, constants and NULLs are already values and can thus be returned as such. The evaluation of term sequences , given an environment, returns the tuple of values corresponding to each of the terms and is implemented in the obvious way.

Queries and tables (, ) evaluate to relations whose arity corresponds to the length of their schema $\tau$ (written $\vert \tau \vert$). Existential subqueries evaluate to a non-emptiness test: their evaluation returns a Boolean which is true if, and only if, the query returns a non-empty relation. The evaluation of frames  returns again a relation, whose arity corresponds to the arity of their cross join: this is obtained by flattening $\varGamma '$ and counting its elements; the judgment for generators operates in a similar way. Conditions evaluate to truth values in ${\mathbf {B}}$: in particular, the evaluation of logical values and connectives , , ,  and  exploits the operations btrue, bfalse, band, bor, and bneg provided to the functor by the input module SEM; similarly, atomic predicates are evaluated using the operation sem_bpred, while to evaluate , we first evaluate the condition *c* recursively, obtaining a truth value in ${\mathbf {B}}$, then we pass this value to is_btrue, which returns a bool (even when we are using 3VL), and finally coerce it back to ${\mathbf {B}}$ using the operation of_bool (this construction ensures that  always evaluates to either btrue or bfalse).

As for well-formedness judgments, we prove a weakening lemma:

#### Lemma 4

If  then, for all $\varGamma '$, we have, where $\mathtt {subenv2} : \mathtt {env}~(\varGamma ',\varGamma ) \rightarrow \mathtt {env}~\varGamma$ takes an environment for a context obtained by concatenation and returns its right projection.

### Discussion

To explain the semantics of queries, let us consider the informal definition [13]:where $\eta '$ is defined as the extension of evaluation $\eta$ assigning values $\overrightarrow{V}$ to fully qualified attributes from $\overrightarrow{T : \sigma }$ (in the notation used by [13], $\eta ' := \eta {\mathop {\oplus }\limits ^{\overrightarrow{V}}} \ell (\overrightarrow{T : \sigma })$). This definition operates by taking the semantics of the tables in the  clause (their cartesian product). For each tuple $\overrightarrow{V}$ contained *k* times in this multiset, we extend the environment $\eta$ with $\overrightarrow{V}$, obtaining $\eta '$. If *c* evaluates to $\mathbf {tt}$ in the extended environment, we yield *k* copies of  in the result.

The definition above makes implicit assumptions (particularly, the fact that $\eta$ and $\eta '$ should be good environments for the expressions whose semantics is evaluated), and at the same time introduces a certain redundancy by computing the number *k* of occurrences of $\overrightarrow{V}$ in the input tables, and using it to yield the same number of copies of output tuples.

In our formalization, the semantics above is implemented using abstract relations rather than multisets. While in the paper definition the environment $\eta '$ is obtained by shadowing names already defined in $\eta$, we can dispense with that since we rule out name clashes syntactically, thanks to the use of de Bruijn indices. The implementation uses dependent types and some of the rules use equality proofs to allow premises and conclusions to typecheck: we will not describe these technical details here, and refer the interested reader to the Coq scripts.
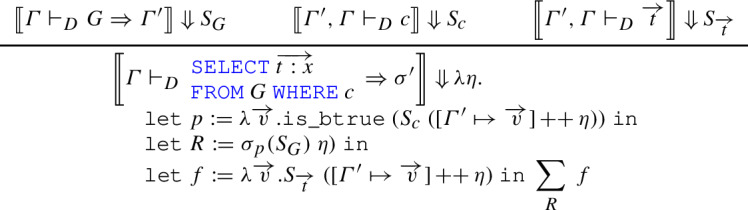


In this mechanized version, the input to the  is generalized to one that may include lateral joins, using  (we get the original version for $n=1$); the relation $R := \sigma _p(S_{G}~\eta )$ replaces the predicate in the multiset comprehension, whereas *f* assumes the role of the output expression. Whenever a certain tuple $\overrightarrow{V}$ appears *k* times in *R*, the relational comprehension operator adds *f* *V* to the output the same number of times, so it is unnecessary to make *k* explicit in the definition. The operation $[\varGamma ' \mapsto \overrightarrow{v}]$ creates an environment for $\varGamma '$ by providing a tuple $\overrightarrow{v}$ of correct length: this constitutes a proof obligation that can be fulfilled by noticing that each $\overrightarrow{v}$ ultimately comes from , whose type is $\mathtt {env}~\varGamma \rightarrow \mathtt {R}~\vert {\mathtt {concat}~\varGamma '} \vert$. Since *G* represents a telescope of lateral joins, its semantics deserves some attention. The interesting case is the following:
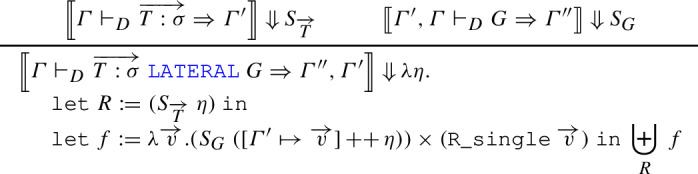


To evaluate a generator  given an environment $\eta$, we first evaluate $\overrightarrow{T:\sigma }$ in $\eta$, obtaining a relation *R*; then, for each tuple $\overrightarrow{v}$ in *R*, we extend $\eta$ with that particular value of $\overrightarrow{v}$ and evaluate *G* recursively in it; we take the product of the resulting relation with the singleton containing the tuple $\overrightarrow{v}$; finally, we perform a disjoint union for all the $\overrightarrow{v}$. Notice that in the absence of  it would have sufficed to perform a product between the semantics of $\overrightarrow{T:\sigma }$ and that of *G*; that is not possible here, because we need to consider a different semantics of *G* for each element of the semantics of $\overrightarrow{T:\sigma }$.

Perhaps a more intuitive way of implementing this semantics would have been a judgment in the form , where $\eta$ is an environment for $\varGamma$ and *R* is the relation resulting from the evaluation of *Q* in that specific environment; however, in the example above, we can see that, in order to compute the relation resulting from the evaluation of the query, the predicate *p* is used to evaluate the condition *c* in various different environments: this forces us to evaluate conditions to functions taking as input an environment, and due to the mutual definition of conditions and queries, the evaluation of queries must result in a function as well.

The appendix contains the full definition of the semantics we formalized. We only consider here the judgment used to evaluate  conditions, as it deserves a brief explanation:



The membership condition must bridge the gap between the three-valued logic of SQL and the Boolean logic used by abstract relations: in particular, to check whether a tuple $\overrightarrow{t}$ appears in the result of a query *Q*, we cannot simply evaluate $\overrightarrow{t}$ to $\overrightarrow{V}$ and *Q* to *S* and check whether $\#(S,\overrightarrow{V})$ is greater than zero, because in three-valued logic NULL is not equal to itself. Instead, given the semantics of *Q*, we compute the number $n^\mathbf {tt}$ of tuples that are equal to $\overrightarrow{V}$ and the number $n^\mathbf {uu}$ of the tuples of *S* that are not different from $\overrightarrow{V}$ (i.e. the matching is up to the presence of some NULLs). If $n^\mathbf {tt}$ is greater than zero, then the condition evaluates to btrue; if $n^ \mathbf {tt}= 0$ but $n^\mathbf {uu}> 0$, the condition evaluates to bmaybe; if both values are zero, then the tuple is certainly not in the result of *Q* and the condition evaluates to bfalse.

The predicates $p^\mathbf {tt}$ and $p^\mathbf {uu}$ used in the definition are defined as follows:

$$\begin{aligned} p^\mathbf {tt}&:= \lambda \overrightarrow{V}. \mathtt {fold\_right2}~(\lambda v,w,\mathtt {acc}. \mathtt {acc} \wedge \mathtt {is\_btrue}~(\mathtt {veq}~v~w))~\mathtt {true}~\overrightarrow{V}~(S_Q~\eta ) \\ p^\mathbf {uu}&:= \lambda \overrightarrow{V}. \mathtt {fold\_right2}~(\lambda v,w,\mathtt {acc}. \mathtt {acc} \wedge \lnot \mathtt {is\_bfalse}~(\mathtt {veq}~v~w))~\mathtt {true}~\\&\quad \overrightarrow{V}~(S_Q~\eta ) \end{aligned}$$

Value equality $\texttt {veq : V -> V -> B}$ returns bmaybe when either of the two arguments is NULL, otherwise corresponds to syntactic equality: fold_right2 iterates veq on pairs of values from the two tuples $\overrightarrow{V}$ and $S_Q~\eta$. Although in Boolean logic a predicate is true precisely when it is not false, in tribool the $p^\mathbf {tt}$ and $p^\mathbf {uu}$ may assume different values.

## Validation of Rewrite Rules

Now that we have a formalized semantics of $\textsf {Null}{} \textit{SQL}$, it is a good time to show that it can be used to verify the soundness of some rewrite rules. The two rules we consider allow tables in the  clause of a query to be shuffled, and nested queries to be unnested. In the following statements, given an index *n* and schema $\sigma = x_1,\ldots ,x_k$, we will write $n.\sigma$ as a shorthand for the term sequence $n.x_1,\ldots ,n.x_k$; if $\overrightarrow{u} = u_1,\ldots ,u_k$, we will write  for the simultaneous substitution of $u_i$ for $x_i$, where $i = 1,\ldots ,k$. The symbol $\simeq$ represents heterogeneous equality.

### Theorem 1

Let $\vert {\tau '} \vert = \vert {\sigma _1} \vert + \vert {\sigma _2} \vert$, and $S, S'$ evaluations such thatThen for all $\eta : \mathtt {env}~\varGamma$, we have $S~\eta \simeq S'~\eta$.

### Proof

The proof proceeds by inversion on the derivation of the two semantic judgments; the hypothesis on the length of $\tau '$ is required for the select clause of the second query to be adequate. The goal simplifies to:under the hypotheses $r_1 \simeq r_2$, , , . We prove by functional extensionality that the rhs is equal to , where $\textit{flip}$ is the function that takes a vector of length $\vert {\sigma _2} \vert +\vert {\sigma _1} \vert$ and swaps the first $\vert {\sigma _2} \vert$ elements with the last $\vert {\sigma _1} \vert$. Then the goal becomes , which is easily obtained by inversion on  and . $\square$

### Theorem 2

Let $S, S'$ be evaluations such thatThen for all $\eta : \mathtt {env}~\varGamma$, we have $S~\eta \simeq S'~\eta$.

### Proof

By inversion on the derivation of the two evaluations (and also using Lemma 3), we know that , , , , .

The lhs of the thesis computes to an abstract expression containing two nested $\sum$ operations; we prove the general result that $\sum _{\sum _r~f}~g = \sum _r~(g \circ f)$ and obtain the new lhs:where $p_c(\overrightarrow{w}) ) := S_c~([\sigma _2 \mapsto \overrightarrow{w}]\mathop {++}\eta )$. The rhs of the goal computes to:Then, for the lhs and rhs to be equal, we only need to prove the following:This is a property of substitution that we prove by induction on the sequence of terms $\overrightarrow{t}$. $\square$

## Elimination of Three-Valued Logic

We now move to formalizing Guagliardo and Libkin’s proof that SQL has the same expressive power under Boolean and three-valued logic, in the sense that for every query evaluated under 3VL, there exists another query with the same semantics in Boolean logic, and vice-versa. The proof is constructive: we exhibit an (algorithmic) transformation $(\cdot )^\mathbf {tt}$ which turns a query for 3VL-SQL into Boolean-SQL (a much simpler transformation $(\cdot )^*$ operates in the opposite direction). The transformation $(\cdot )^\mathbf {tt}$ is defined by mutual recursion on queries, tables, and conditions; more precisely, $(\cdot )^\mathbf {tt}$ is mutually defined with an auxiliary transformation $(\cdot )^\mathbf {ff}$, operating on conditions only: the rationale is that while $c^\mathbf {tt}$ is true in Boolean logic when *c* is ttrue in 3VL, $c^\mathbf {ff}$ is true in Boolean logic when *c* is tfalse in 3VL; as a corollary, when *c* evaluates to 3VL unknown, both $c^\mathbf {tt}$ and $c^\mathbf {ff}$ are Boolean false.

**Fig. 4:**
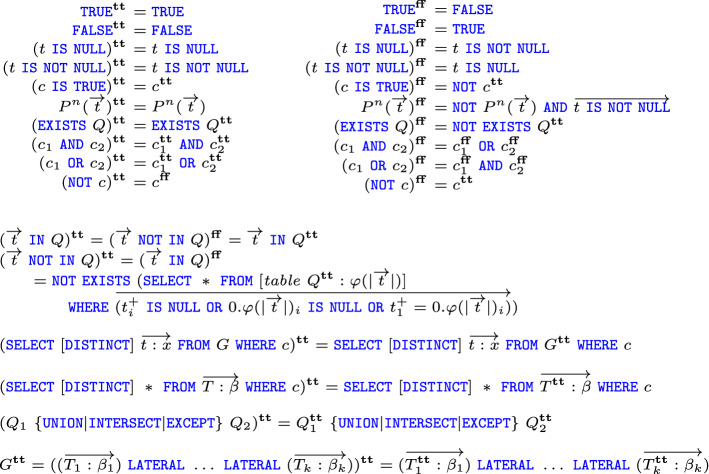
Translation from 3VL-SQL to 2VL-SQL

Figure 4 shows the definition of these transformations: these extend Guagliardo and Libkin’s version by adding cases for  query inputs and for the  test. Most of the interesting things happen within conditions: while the definition of ()$^\mathbf {tt}$ simply propagates the transformation to the nested query, the definition of ()$^\mathbf {tt}$ is more involved: it requires us to evaluate $^\mathbf {tt}$ as a nested query and then keep those tuples that are equal to $\overrightarrow{t}$ up to the presence of s (either in $\overrightarrow{t}$ or in ); if the resulting relation is not empty, the condition evaluates to true; in the formalization a fold_right operation is used to generate all the conditions on the elements of $\overrightarrow{t}$ and of the tuples from . The definition of this case is further complicated by the fact that the schema of *Q* may not be well-formed, so we need to replace it with a new schema made of pairwise distinct names (generated on the fly by the $\varphi$ operation); furthermore, since in the translated query we use $\overrightarrow{t}$ inside a nested  query (thus, in an extended context), we use the tm_lift operation to increment the de Bruijn indices it may contain (in the figure, we use the notation $t^+_i$ for this operation). Negations are translated as ()$^\mathbf {tt}= c^\mathbf {ff}$; the transformation commutes in the other cases.

As for the negative translation $(\cdot )^\mathbf {ff}$, it proceeds by propagating the negation to the leaves of the conditional expression (using de Morgan’s laws for s and s). The membership tests ()$^\mathbf {ff}$ and ()$^\mathbf {ff}$ are defined as in the positive translation, but with their roles swapped. In the interesting case, we translate $P^n(\overrightarrow{t})^\mathbf {ff}$ by checking that $P^n(\overrightarrow{t})$ is not true and that all elements of $\overrightarrow{t}$ are not null (here as well, the condition is computed by means of a fold_right on the elements of $\overrightarrow{t}$). The two translations are described by the following Coq code. 
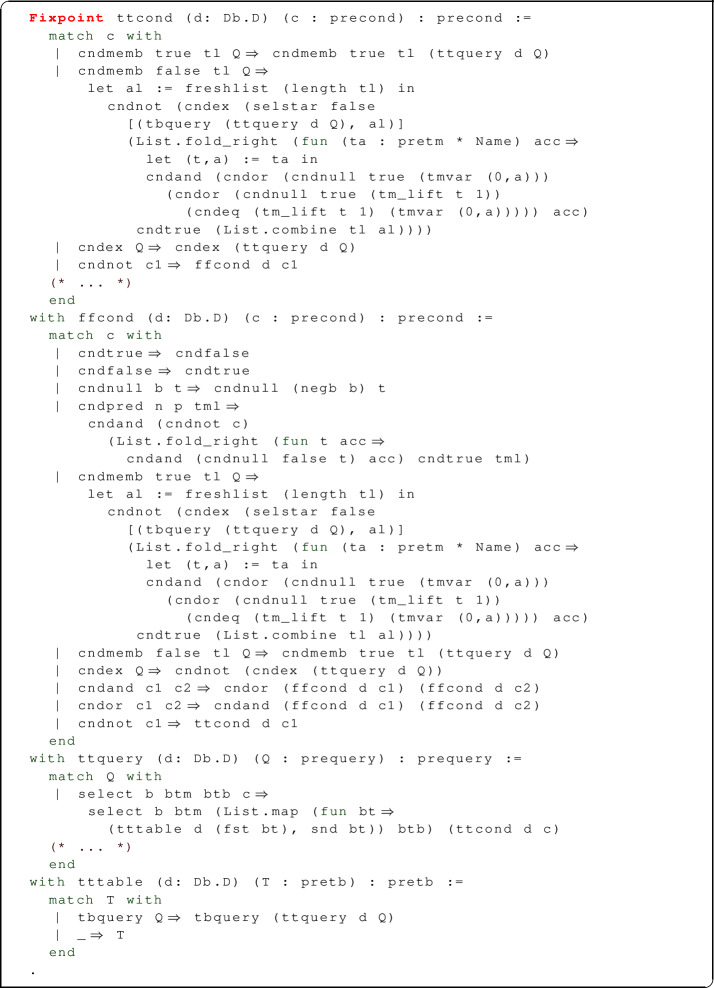
 We prove that the translation preserves the semantics of queries in the following theorem.

### Theorem 3

For all queries *Q*, if , there exists $S'$ such that

 and for all $\eta : \mathtt {env}~\varGamma$, $S~\eta = S'~\eta$.

The proof of the theorem is by induction on the semantic judgments yielding *S*: this is actually a mutual induction on the five mutually defined evaluations. For the part of the proof that deals with conditions, we need to prove a stronger statement that essentially says that $c^\mathbf {tt}$ evaluates to true only if *c* evaluates to ttrue, and $c^\mathbf {ff}$ evaluates to true only if *c* evaluates to tfalse: in other words, $c^\mathbf {tt}$ asserts the truth of *c*, while $c^\mathbf {ff}$ asserts its falsehood.

An immediate question raised by this result asks whether a realistic semantics for $\textsf {Null}{} \textit{SQL}$ can be derived from a semantics that does not have a special treatment of null values, just by translating input queries under the the $(\cdot )^\mathbf {tt}$ transformation. The answer is affirmative in principle: however, to prove the validity of rewrite rules under that semantics, one would then need to reason not on the original query *Q*, but on its translated version $Q^\mathbf {tt}$. This would greatly complicate the proof since, recursively, one would need to reason on conditions using two different induction hypotheses for their positive and negative translation.

## Embedding the Relational Calculus

We now formalize a relational calculus to show that its normal forms can be translated to SQL in a semantically preserving way. The calculus we describe is a variant of the heterogeneous nested relational calculus ( [20, 21]), which provides both set and bag semantics, enriched with a constant  to account for indeterminate values. All variants of  allow terms of nested collection type, which cannot be expressed in SQL directly; however, we will show that normal forms whose type is a flat relation can be translated to SQL.

The terms of  are defined by the following grammar:Variables are represented as de Bruijn indices *n*. The grammar provides empty collections and singletons, along with the standard operations of union, intersection, and difference; empty collections $\emptyset$ and singletons $\{{M}\}$ are annotated with a subscript *b* representing their kind, which can be $\mathsf {set}$ or $\mathsf {bag}$; empty collections are additionally annotated with their schema $\sigma$; the other collection operations do not require annotations. There are also operations $\delta$ and $\iota$, which, respectively, convert a bag into a set by duplicate elimination, and promote a set to a bag in which each element has multiplicity equal to 1. A comprehension  binds a variable in $M_1$: semantically, this corresponds to the union of the $M_1[V/0]$ for all values *V* in the collection $M_2$ (this is a set or bag union depending on whether $M_1$ and $M_2$ are sets or bags); $M_1$ and $M_2$ are called the head and the generator of a comprehension, respectively. The one-armed conditional  is equivalent to $M_1$ when $M_2$ is true, and to an empty collection otherwise. The emptiness test $\mathbf {empty}_b(M)$ is annotated with a Boolean depending on whether its argument is a set or a bag.

Tuples with named fields , and tuple projections *M*.*x* are standard; null values , constants ${\mathbf {k}}$, standard Boolean operations and constants, the test for nullness , the test for truth , custom predicates $P^n(\overrightarrow{M_n})$, and table references $\textit{table}~x$ are similar to the corresponding SQL concepts of Sect. 3.

**Fig. 5:**
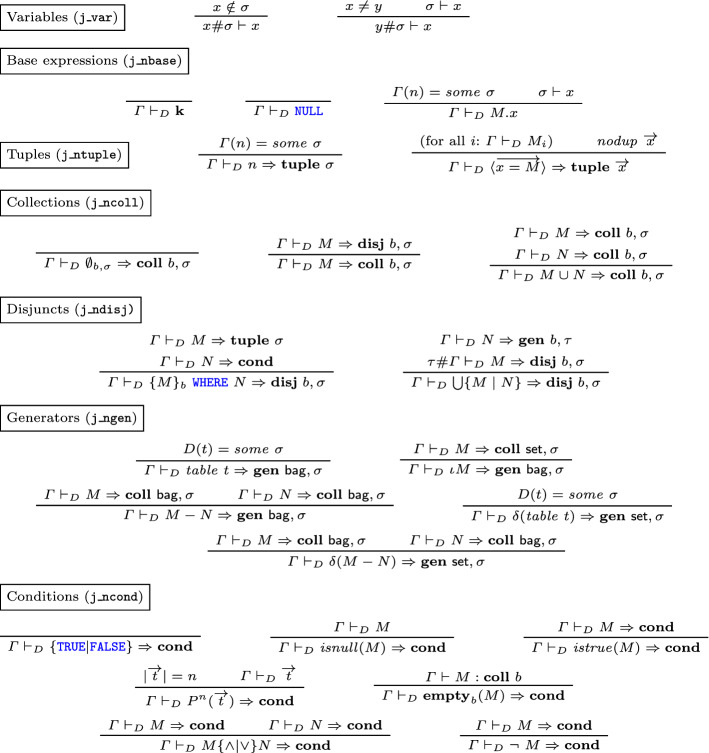
Relational Calculus normal forms

The abstract syntax above corresponds to the following Coq implementation. 
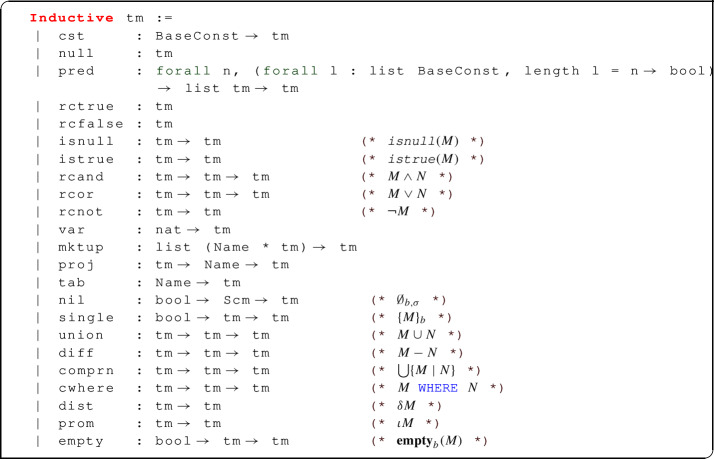
 The most important difference between this concrete syntax and the abstract one is that where the latter uses subscripts $\mathsf {bag}$, $\mathsf {set}$, the former employs a Boolean which is true for sets, and false for bags. In this formalization, we are only interested in assigning meaning to RC *normal forms*, corresponding to the terms in this grammar:In Coq, we define normal forms by means of an inductive judgment described in Fig. 5. Similarly to the grammar, the judgment partitions normal forms in various categories depending on their type: base expressions, tuples with a certain schema $\sigma$ ($\mathbf {tuple}~\sigma$), conditional tests ($\mathbf {cond}$), and collections of tuples ($\mathbf {coll}~b,\sigma$), where *b* can be $\mathsf {bag}$ or $\mathsf {set}$. Collections in normal form are defined as unions of nested comprehensions, thanks to auxiliary categories $\mathbf {disj}~ b,\sigma$ and $\mathbf {gen}~ b,\sigma$ representing, respectively, comprehensions and comprehension generators.

### Semantics

**Fig. 6:**
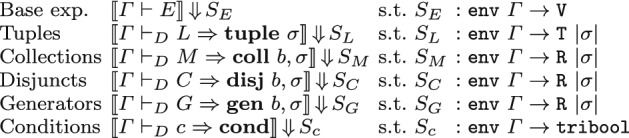
Formal semantics of the Relational Calculus (types)

We provide semantic evaluation judgments for RC terms using the same approach we presented in Sect. 5 for SQL queries: as shown in Figure 6, there is a separate judgment for each of the syntactic categories of terms in normal form. All terms are interpreted using 3VL rather than Boolean logic.

The evaluation of a base expression maps an environment to a value; valuations of sequences of base expressions return tuples of values, with arity corresponding to the length of the sequence; similarly, the evaluation of an RC tuple returns a tuple of values, with arity corresponding to the length of the tuple schema. Collections (and the auxiliary categories of disjuncts and generators) are mapped to evaluations returning relations, whose arity matches the schema of the input expression. Finally, the evaluation of conditions returns a truth value from $\mathtt {tribool}$.

Simple attributes are defined in a schema rather than a context: their semantics  maps an environment for the singleton context $[\tau ]$ to a value. Similarly, the semantics of fully qualified attributes  maps an environment for $\varGamma$ to a value. In both cases, the output value is obtained by lookup into the environment.

The evaluation of terms  returns a value for *t* given a certain environment $\gamma$ for $\varGamma$. In our definition, terms can be either full attributes *n*.*x*, constants ${\mathbf {k}}$, or NULL. We have just explained the semantics of full attributes; on the other hand, constants and NULLs are already values and can thus be returned as such. The evaluation of term sequences , given an environment, returns the tuple of values corresponding to each of the terms and is implemented in the obvious way.

### Conversion to SQL

**Fig. 7:**
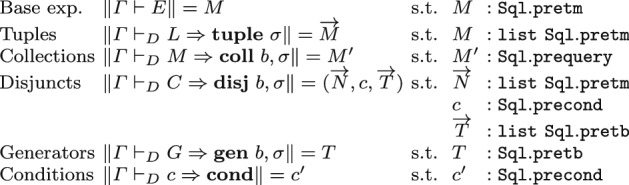
Relational Calculus translation to SQL (types)

**Fig. 8:**
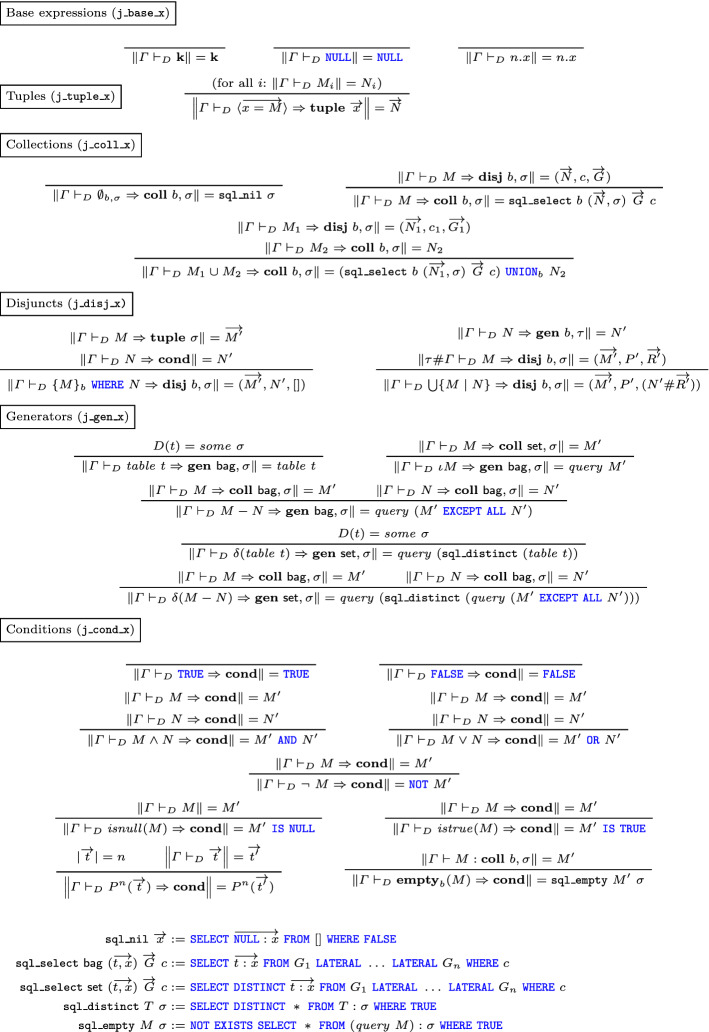
Relational Calculus translation to SQL

Finally, in Figure 7 and 8, we formalize type and definition of the translation of normal form RC terms to SQL expressions: just like the RC semantics, this definition comprises several mutually inductive judgments, following the structure of normal forms rather than that of general RC expressions: this allows us to translate base expressions to SQL terms, tuples to sequences of SQL terms, conditions to SQL conditions, and collections to SQL queries. Comprehension generators are translated to SQL tables (which can be database tables or inner queries to be used in the  clause of an external query). Finally, disjuncts must return the three clauses of a  statement: these are returned separately as a triple (for technical reasons related to the fact that recursion is needed to collect all these items in the case of nested comprehensions), and it is up to the collection translation judgment to compose them into a single SQL statement.

The translation rules use some additional definitions as useful shorthands: $\mathtt {sql\_nil}$ returns an SQL query returning an empty relation of a certain schema; $\mathtt {sql\_select}$ composes its input into a  statement: an important point to note is that all the inputs to this query are declared as  due to the fact that the in the relational calculus, in a nested comprehension of the form $\bigcup \{{\bigcup \{{L \mid M}\} \mid N}\}$, *M* is allowed to reference the tuples in *N*: therefore, similar dependencies must be allowed in the output of the translation as well. Another auxiliary definition $\mathtt {sql\_distinct}$ uses  to deduplicate an input table with a given schema; $\mathtt {sql\_empty}$ constructs an SQL condition which is true whenever a certain query evaluates to an empty relation.

We are able to prove that the translation above is correct, by showing that the semantics of an RC collection expression is equal to that of the corresponding SQL query:

#### Theorem 4

Suppose ; then, for all $M'$ such that , there exists $S_{M'}$ such that  and for all $\eta : \mathtt {env}~\varGamma$, we have $S_M~\eta = S_{M'}~\eta$.

The proof of the theorem is by induction on the semantic judgment yielding $S_M$, followed by inversion on the translation of *M* to $M'$. More precisely, the proof uses mutual induction on the four mutually defined judgments for the semantics of collections, disjuncts, generators, and conditions.

## Related Work

### Semantics of Query Languages with Incomplete Information and Nulls

Nulls arise from the need for *incomplete information* in databases, which was appreciated from an early stage. Codd [7] made one of the first proposals based on null values and three-valued logic, though it was criticized early on due to semantic irregularities and remains a controversial feature [11, 23]. A great deal of subsequent research has gone into proposing semantically satisfying approaches to incomplete information, in which a database with null values (or other additional constructs) is viewed as representing a *set of possible worlds*, and we wish to find *certain* query answers that are true in all possible worlds. Many of these techniques are surveyed by van der Meyden [24], but most such techniques either make query answering intractable (e.g. coNP-hard), have semantic problems of their own, or both. However, SQL’s standard behaviour remains largely as proposed by Codd, leading database researchers such as Libkin [16] to propose revisiting the topic with an eye towards identifying *principled* approaches to incomplete information that are *realistic* relative to the standard capabilities of relational databases. For example, Libkin [17] compares certain answer semantics with SQL’s actual semantics, shows that SQL’s treatment of nulls is neither sound nor complete with respect to certain answers, and proposes modifications to SQL’s semantics that restore soundness or completeness while remaining (like plain SQL) efficiently implementable.

Some work has explored the semantics and logical properties of nulls in set-valued relational queries, but did not grapple with SQL’s idiosyncrasies or multiset semantics [9]. Guagliardo and Libkin [13] were the first to define a semantics that is a realistic model of SQL’s actual behaviour involving both multisets and nulls. They empirically validated a (Python) implementation of the semantics against the behaviour of real database systems such as PostgreSQL and MySQL, and confirmed some minor but nontrivial known discrepancies between them in the process. In addition they gave (paper) proofs of the main results relating the SQL semantics, three-valued and two-valued semantics. Our work complements and deepens this work by making all notions of their semantics precise and formal, and formally proving their main result relating the three-valued and two-valued semantics.

Because our formalization follows Guagliardo and Libkin’s on-paper presentation closely, it benefits indirectly from their extensive experimental validation. Nevertheless, there remains a small “formalization gap” between our work and theirs in the sense that our (formally validated) Coq definitions might differ from their (empirically validated) Python implementation. So, in addition to extending the coverage of SQL features as discussed below, it could be worthwhile to derive an executable semantics from our definitions and empirically validate it against the same examples they used.

#### Formalizations of Query Languages

Malecha et al. [18] formalized components of a relational database engine (including a front-end providing a SQL-like relational core, optimization laws including side-conditions, and an implementation of B+-trees) in Coq using the YNot framework. Their work (like most prior formalizations) employs set semantics; while the data model allows for fields to have optional types, the behaviour of missing values in primitive operations is not discussed, and their semantics is the standard two-valued, set-theoretic interpretation of relational algebra. The main technical challenge in this work was verifying the correctness of imperative algorithms and pointer-based data structures used in efficient database implementations. Benzaken et al. [3] formalized the relational data model, going beyond the core relational operations in Malecha et al.’s formalization to include integrity constraints (functional dependencies). They formalize a number of algorithms from database theory whose standard presentations are imprecise, and showed that careful attention to variable binding and freshness issues is necessary to verify them. Their formalization included proofs of correctness of relational rewrite rules (with respect to the set-theoretic semantics) but did not directly consider SQL queries, multiset semantics, or features such as nulls.

Chu et al. [6] presented a new approach to formalizing and reasoning about SQL, called $\textsf {HoTT}{} \textit{SQL}$. $\textsf {HoTT}{} \textit{SQL}$ uses homotopy type theory to formalize SQL with multiset semantics, correlated subqueries, and aggregation in Coq. $\textsf {HoTT}{} \textit{SQL}$ is based on the intriguing insight (inspired by work on semiring-valued database query semantics [12]) that we can define multisets as *functions* mapping tuples to cardinalities. They propose representing cardinalities using certain (finite) *types* thanks to the univalence axiom; this means that Coq’s strong support for reasoning about types can be brought to bear, dramatically simplifying many proofs of query equivalences. However, since $\textsf {HoTT}{} \textit{SQL}$ does not consider nulls or three-valued logic, it validates query equivalences that become unsound in the presence of nulls. Unfortunately, it does not appear straightforward to extend the $\textsf {HoTT}{} \textit{SQL}$ approach of conflating types with semiring annotations to handle SQL-style three-valued logic correctly. In addition, the adequacy of $\textsf {HoTT}{} \textit{SQL}$ ’s approach requires proof. It should also be noted that the univalence axiom used by homotopy type theory and Streicher’s K axiom required to work with John Major equality, which we used in our formalization, are incompatible: this would make it challenging to merge the two efforts.

Most recently, Benzaken and Contejean [2] proposed a formal semantics for a subset of SQL ($\textit{SQL}_{\textit{Coq}}$) including all of the above-mentioned features: multiset semantics, nulls, grouping and aggregation. SQL has well-known idiosyncrasies arising from interactions among these features: for example, the two queries 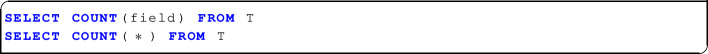 are not equivalent. The first one counts the number of *non-null* values in *T*, while the second counts the number of rows, ignoring their (possibly null) values. These two queries *are* provably equivalent in the $\textsf {HoTT}{} \textit{SQL}$ semantics, but are correctly handled by $\textit{SQL}_{\textit{Coq}}$.

Moreover, Benzaken and Contejean highlight the complexity of SQL’s treatment of grouping and aggregation for *nested subqueries*, propose a semantics for such queries, and prove correctness of translations from $\textit{SQL}_{\textit{Coq}}$ to a multiset-valued relational algebra $\textit{SQL}_{\textit{Alg}}$. Their work focuses on bag semantics and uses a Coq library for finite bags, and treats duplicate elimination as a special case of grouping. While grouping can be expressed, in principle, by desugaring to correlated subqueries (an approach proposed by Buneman et al. [4] and adopted by $\textsf {HoTT}{} \textit{SQL}$, which we could also adapt to our setting) these features of $\textit{SQL}_{\textit{Coq}}$ highlight many intricacies of the semantics of grouping that make it difficult to get such a desugaring right.

We can highlight several aspects where our work complements $\textit{SQL}_{\textit{Coq}}$: (1) superficially, their approach does not deal with named aliases for table records, requiring additional renaming; (2) their novel semantics is tested on example queries but not evaluated as thoroughly as Guagliardo and Libkin’s; (3) we present well-formedness criteria for $\textsf {Null}{} \textit{SQL}$, which are more accurate than those considered for $\textit{SQL}_{\textit{Coq}}$, ensuring that queries with unbound table references should not be accepted; (4) their work does not consider formal results such as the equivalence of 2-valued and 3-valued semantics, which to the best of our knowledge has not been investigated in the presence of grouping and aggregation; (5) the fragment of SQL formalized in our work allows lateral joins, an SQL:1999 feature that is becoming increasingly popular thanks to the support by recent versions of major DBMSs; (6) building on the support for lateral joins, we are able to formalize a verified translation from the nested relational calculus to SQL, which is of interest for the theory of programming languages supporting language-integrated query. Finally, because of the complexity of their semantics (required to handle SQL’s idiosyncratic treatment of grouping and aggregation), our formalization may be preferable for proving properties of queries that lack these features; it would be enlightening to formally relate our formalization with theirs, and establish whether equivalences proved in $\textsf {Null}{} \textit{SQL}$ are still valid in $\textit{SQL}_{\textit{Coq}}$.

Formalization has also been demonstrated to be useful for designing and implementing new query languages and verified transformations, for example in the QCert system [1]. This work considers a nested version of relational calculus, and supports a subset of SQL as a source language, but does not appear to implement Guagliardo and Libkin’s semantics for SQL nulls. It could be interesting to incorporate support for SQL-style nulls into such a verified query compiler.

## Conclusion

We have mechanically checked the recently proposed semantics of $\textsf {Null}{} \textit{SQL}$ [13] and proved the main results about its metatheory. Our work should be compared to two recent formalizations, $\textsf {HoTT}{} \textit{SQL}$ [6], and $\textit{SQL}_{\textit{Coq}}$ [2]. Compared to $\textsf {HoTT}{} \textit{SQL}$, our representation of multisets is elementary and it does not appear straightforward to adjust $\textsf {HoTT}{} \textit{SQL}$ to handle null values, since its treatment of predicates using homotopy type theory assumes standard two-valued logic. Compared to $\textit{SQL}_{\textit{Coq}}$, our semantics is simpler and closely modeled on the on-paper semantics of [13], which was thoroughly tested against real database implementations. Our work is also the first formalization of SQL to consider queries with lateral inputs. On the negative side, compared to both $\textsf {HoTT}{} \textit{SQL}$ and $\textit{SQL}_{\textit{Coq}}$, our formalization does not attempt to handle grouping and aggregation, but as a result it may be simpler and easier to use, when these features are not needed.

In this paper we also presented the first ever mechanized proofs of the expressive equivalence of two-valued and three-valued SQL queries, the first ever verified translation of relational calculus queries to SQL queries, and the correctness of rewrite rules that are valid for SQL’s real semantics (including multisets and nulls). The diversity of recent approaches to formalizing SQL also suggests that consolidation and cross-fertilization of ideas among approaches may reap rewards, to provide a strong foundation for exploring verification of other key components of database systems.

## Commented Semantics of $\textsf {Null}{} \textit{SQL}$

We give here a commented version of the formalized semantics of $\textsf {Null}{} \textit{SQL}$ beyond what was possible to report on in the paper. The semantics consists of four inductive judgments for simple attributes, full attributes, terms and terms sequences (j_var_sem, j_fvar_sem, j_tm_sem, j_tml_sem), and five mutually defined judgment for the main SQL expressions, namely queries (j_q_sem), tables (j_tb_sem), conditions (j_cond_sem), table bindings (j_btb_sem), and existentially nested queries (j_in_q_sem).

### Semantics of Attributes

An attribute is evaluated in a singleton context [*s*] under an environment for that context.

 Under a context *a* :  : *s*, the semantics of *a* is the head value in the environment; we also check that *a* should not be in the remainder of the context for well-formedness. Under a context *a* :  : *s* where $a \ne b$, we first evaluate *b* under the remainder context *s* and lift the resulting evaluation from context *s* to context *a* :  : *s*.

The judgment for full variables (in the form *n*.*a*, where *n* is a de Bruijn index) lifts the semantics of simple variables to contexts composed of multiple schemas.

 To evaluate attributes in the form 0.*a* in a context *s* :  : *G*, we first evaluate the simple attribute *a* in *s* and then lift the resulting evaluation from [*s*] to *s* :  : *G*. The evaluation of $(i+1).a$ is obtained recursively by evaluating *i*.*a* in *G* and lifting the valuation to *s* :  : *G*.

### Semantics of Terms

A term *t* is evaluated in a context $\varGamma$ to a function from a suitable environment to values.

 While the semantics of constants and nulls is trivial, full variables are evaluated in the judgment for full variables.

The evaluation of sequences of terms is similar, but it returns a tuple of values of corresponding size.

 This judgment is implemented in the obvious way, by mapping empty sequences of terms to an evaluation returning the empty tuple, and by recursion when the input list of terms is not empty.

### Semantics of Queries

If a query *Q* with schema $\sigma$ is evaluated in a context $\varGamma$, we obtain a function returning a relation with arity corresponding to $\sigma$.

 The evaluation of select queries was described in detail in the paper. Here, we just notice that the list tml contains pairs of terms and attribute names, where attribute names are used to produce the output schema. Since the Coq typechecker cannot automatically infer that the arity of the semantics for the list of terms List.map fst tml matches the arity of the schema List.map snd tml, the rule takes evidence of this fact in the form of an equation e. The output relation is flattened to a set relation if the  clause (signaled by the boolean b) was used.

The evaluation of select star queries proceeds similarly, by desugaring the star to a list of terms (tmlist_of_ctx G0). ,  and  queries are implemented all in the same fashion, by evaluating their subqueries recursively and combining them with the relational operators $\oplus$, $\cap$ and $\setminus$ from the ADT.

When a query *Q* is evaluated in a context $\varGamma$ as an existentially nested query, we obtain a function returning a Boolean denoting whether the resulting relation is non-empty.

 The implementation of the semantics of existentially nested queries mostly reflects, in a simplified way, the corresponding rules for general queries. At the end, the resulting relation is tested for non-emptiness by checking whether its cardinality is greater than zero or not.

### Semantics of Tables

The type of the semantics of tables is similar to that of the semantics of queries.

 The definition is trivial: the data base provides semantics for stored named tables, whereas tables resulting from queries are evaluated by means of their judgment.

The type of the semantics of frames (sequences of tables) is as follows:

 The sequence of tables is unfolded as in the case of terms. The base case for empty sequences returns the 0-ary relation Rone, which is the neutral element for the cartesian product of relations; non null sequences are evaluated recursively, and the resulting semantics are combined by means of the relational operator $\times$. A cast is used to make the definition typecheck.

The type of the semantics of generators (lateral joins of frames) is as follows:

 The generator too is evaluated one frame at a time, ending with the 0-ary relation Rone in the case of the empty sequence of frames: however, unlike the evaluation of simple frames, in a generator we do not perform a simple cartesian product of the semantics of the components, because a certain frame may depend on the ones declared to its left. More details are provided in the paper.

### Semantics of Conditions

The evaluation of conditions returns a truth value in the abstract type ${\mathbf {B}}$.

 The evaluation of  and  is trivial, returning the corresponding elements of type ${\mathbf {B}}$. To evaluate , we first evaluate t and then check whether the evaluation returns null or not. Similarly, to evaluate , we first evaluate c and then check whether the evaluation yields  or not.  This is the evaluation of an n-ary basic predicate p applied to a list of terms tml. We first obtain Stml as the evaluation function for tml, then the evaluation for the basic predicate is a function that takes an environment Vl as input and returns the result of p applied to (Stml Vl). However, p expects to receive list of constants, while (Stml Vl) is a tuple that may contain nulls: so, we first convert the tuple to a list, and then we use the operation sem_bpred from the ADT for B to lift a predicate of type $\texttt {list BaseConst -> bool}$ to one of type $\texttt {list Value -> B}$.  The evaluation of membership of a tuple within a nested query was discussed in the paper; in the concrete definition, the boolean b is used to differentiate between IS IN Q and IS NOT IN Q. Casts are also added to make the definitions typecheck.  conditions are implemented by the existentially nested query judgment $\texttt {j\_in\_q\_sem}$. The remaining conditions implement logical connectives by means of the corresponding operations on the ADT of truth values.

## Commented Semantics of the Flat Relational Calculus

### Semantics of Base Expressions

The semantics of base expressions of flat relational terms in normal form has the following type:

Sequences of base terms are also given a semantic evaluation judgment for convenience:

 In the relational calculus, base expressions serve the same purpose as SQL terms, and their semantics are analogous.

### Semantics of Tuples

The semantics of flat relational calculus tuples in normal form has the following type:

 Normal forms of tuples are sequences of pairs attribute name-base expression (List.combine al bl): their semantics is obtained by evaluating the sequence of base expressions (bl), with a cast to make the judgment typecheck.

### Semantics of Conditions

The type of the semantics of conditions is as follows:

 The semantics of the emptiness test on a collection q first evaluates q recursively, and then uses an auxiliary definition sem_empty that checks whether the resulting relation has cardinality equal to zero.  The remaining cases of the semantics of relational calculus conditions closely correspond to $\textsf {Null}{} \textit{SQL}$ conditions, and their semantics is similar.

### Semantics of Collections

The type of the semantics of collections is as follows:

 To evaluate nil b s (that is, an empty collection with schema s, where the Boolean b specifies whether the collection is a set or a bag), we need to provide an empty relation with arity equal to the length of s: this is returned by the function sem_nil, which first builds a singleton containing a tuple of nulls of suitable length, and then filters it using the trivially false predicate. If the collection is a disjunct, it is evaluated by a separate judgment; if it is a union union t1 t2 (where t1 is a disjunct and t2 a collection), the two subterms are evaluated recursively and then their semantics are combined using Rel.plus (this is followed by a call to Rel.flat to perform deduplication if b is true, signalling the collection is a set).

### Semantics of Disjuncts

The type of the semantics of disjuncts is the following:

 A disjunct is either , where *M* is a tuple and *N* is a condition, or a comprehension whose head is a disjunct. In the first case, we evaluate *M* and *N* using the respective judgments: if *N* evaluates to true, we use Rel.Rsingle to return a singleton relation containing a tuple corresponding to the semantics of *M*; otherwise we return an empty relation of appropriate arity using sem_nil.

In the case of a comprehension $\bigcup \{{M \mid N}\}$, we first evaluate the generator *N*, then for each element $\overrightarrow{v}$ of the resulting relation we evaluate the semantics of *M* in an environment extended with $\overrightarrow{v}$; finally, we take the take the disjoint union of all the resulting relations using Rel.rsum (this is followed by a deduplication step if we are evaluating a set rather than a bag.

### Semantics of Generators

The type of the semantics of generators is the following:

 The evaluation of named tables is provided by the database through Db.db_rel.  A bag generator can be a set collection q promoted to a bag. Its semantics is trivial, resorting to a recursive evaluation of q as a set collection.  A generator consisting of the bag difference between two collections q1 and q2 is evaluated by taking the semantics of q1 and q2 as collections, and using Rel.minus to obtain the relation corresponding to their difference.  The semantics of deduplicated tables and bag differences is similar to the non-deduplicated case, but uses Rel.flat to deduplicate the result.
